# The spatial organization of craft production at the Kura-Araxes settlement of Köhne Shahar in northwestern Iran: A zooarchaeological approach

**DOI:** 10.1371/journal.pone.0229339

**Published:** 2020-03-04

**Authors:** Siavash Samei, Karim Alizadeh

**Affiliations:** 1 Humanities Institute, University of Connecticut, Storrs, Connecticut, United States of America; 2 Department of Anthropology, Grand Valley State University, Allendale, Michigan, United States of America; University at Buffalo - The State University of New York, UNITED STATES

## Abstract

The Kura-Araxes cultural tradition (ca. 3500–2200 BCE) was one of the most widespread archaeological horizons in Southwest Asian prehistory, spanning from the Caucasus to the southern Levant. Although several decades of research have considerably increased our knowledge about this Early Bronze Age tradition, the social and economic organization of its communities remains a matter of much debate. Interpretations of the organization of Kura-Araxes craft economies range from need-based household production to extra-household specialized production for exchange and elite consumption. This issue stems from the absence of a systematic approach to studying the spatial organization of craft production; that is the study of the spatial distribution of archaeological material across multiple contexts to reconstruct the location of various activities involved in craft production. Extensive evidence for craft production at Köhne Shahar (KSH)—one of the largest Kura-Araxes sites ever discovered—provides an opportunity for such a study. Faunal remains are among the most abundant types of remains recovered at KSH Phases IV and V (ca. 2800–2500 BCE), where bone and antler were cached and then shaped into tools that were used to manufacture other objects like beads. We use zooarchaeology to study the spatial distribution of worked and unworked animal remains to analyze the function of several structures and spaces in the craft production areas to ultimately gain better insight into the organization of labor and the social organization of the community. We identify evidence for two antler storage units, numerous waste dumps, and several workshops. Some workshops manufactured a similar range of small, possibly ornamental objects, while others specialized in the manufacture of certain goods, textiles, and objects made of animal horn. When combined with other lines of evidence, our observations point to a community-wide production economy with little direct evidence for a stratified social organization. We argue that the absence of a strict social hierarchy at KSH and across the Kura-Araxes world is not evidence of the absence of social complexity, but the presence of a horizontal or heterarchical social order.

## Introduction

**Fig 1:**
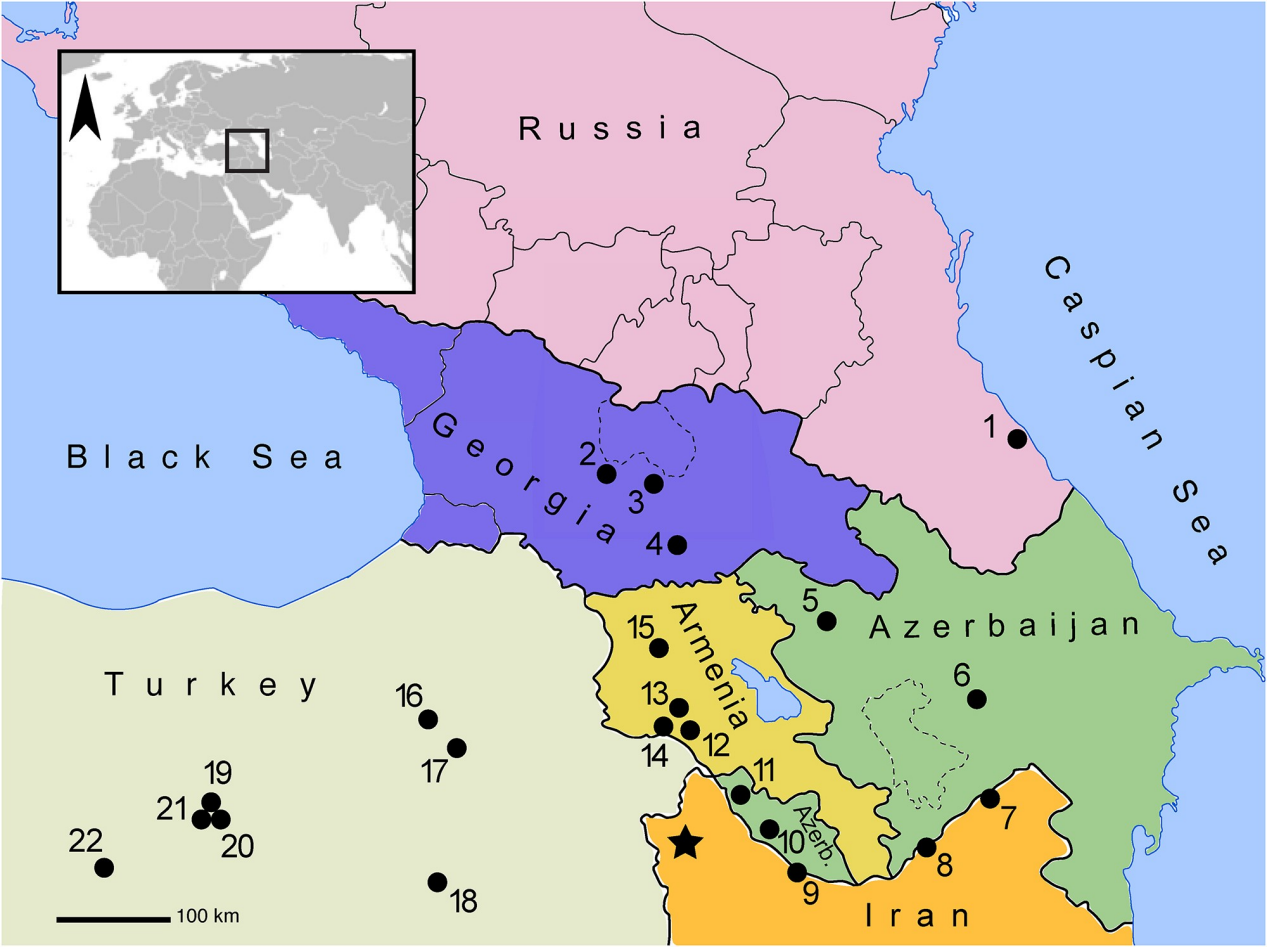
Location of Köhne Shahar (KSH) and other major Kura-Araxes sites in the southern Caucasus and Anatolia, including those discussed in this paper. Republished from Wikimedia Commons under a CC BY license, with permission from Pmx and Romaine, original copyright 2014. KSH is marked by a star. 1. Velikent, 2. Kvatskhelebi, 3. Natsagora, 4. Balitshi-Dzedzvebi, 5. Mentesh Tepe, 6. Leilatepe, 7. Nadir Tepesi, 8. Köhné Pāsgāh Tepesi, 9. Kül Tepe Jolfa, 10. Duzdaği, 11. Ovçular Tepesi, 12. Jrashen, 13. Shengavit, 14. Mokhra Blur, 15. Gegharot, 16. Sos Höyük, 17. Tepecik, 18. Muş, 19. Korucutepe, 20. Tülintepe, 21. Norşuntepe, 22. Arslantepe.

This study investigates the spatial distribution of worked and unworked faunal remains to analyze the function of various structures and spaces at the Kura-Araxes settlement of Köhne Shahar to ultimately gain better insight into the organization of craft production. The Kura-Araxes (KA) cultural tradition was arguably the most geographically-widespread and long-lived archaeological tradition in Southwest Asian prehistory [1,2]. This tradition first emerged in the highlands of the southern Caucasus in the mid-fourth millennium BCE (Fig 1). By the early third millennium BCE, it had expanded beyond its southern Caucasus “homeland.” Kura-Araxes “diaspora” communities appeared as far west as Anatolia and the southern Levant, as far south as the central Zagros and the Caspian littoral, and as far north as the edges of the Eurasian Steppe [3–6]. The KA tradition is characterized by a corpus of material culture, including handmade black burnished pottery—sometimes with contrasting color schemes—Nakhichevan lugs, simple arsenical copper or bronze tools and ornaments, and fixed hearths and portable andirons [4,7–10]. Although KA sites are easily identified in the archaeological record [11], the social and economic structures of KA communities remain poorly understood [12].

Data generated over the past two decades attests to considerable heterogeneity in KA economic activities. These activities include farming and animal management, as well as industrial and manufacturing practices such as bead, ceramic, and lithic production; metallurgy; and textile manufacturing [13–17]. Yet how these manufacturing activities were organized remains unclear. Was craft production organized at the household level or was it performed by specialist artisans? Were one or a set of workers able to perform all stages of production or was the labor divided based on specialization? Was the economy organized hierarchically so that production was controlled by an elite apparatus, or did the economy take on a more horizontal structure? Answering these questions requires investigation of the spatial organization of craft production at individual sites [18].

Extensive evidence for craft production at the Köhne Shahar (ca. 3200–2500 BCE) provides an opportunity for such a study at one of the largest KA sites recorded to date. A previous study at Köhne Shahar (KSH) used architecture, artifactual remains, and a geophysical survey to identify extensive evidence for site-wide craft production activities [13]. This evidence centered around several primary workshops where objects including beads were manufactured. This study also identified several waste disposal areas where ash, slags, broken crucibles, and other manufacturing debris were discarded [13]. However, important questions remain about craft production at KSH, including the range of objects manufactured, the tools used in their manufacture, and the purpose of several seemingly empty structures and spaces whose functions are unknown because they lack notable features as well as ceramic, metal, and stone artifacts.

Faunal remains are among the most abundant classes of worked and unworked material at KSH and are found across the aforementioned primary workshops, waste dumps, and “empty” structures. In this study we recorded zooarchaeological data from all faunal remains including both worked and unworked specimens. We then apply a sequence of zooarchaeological analyses to the animal bones to determine how the faunal remains were used at the site—i.e. in food production, food consumption or manufacturing activities. Then, using presence/absence of surface modifications and average fragment size, we divide the faunal remains used for manufacturing into three categories—raw materials, tools, and waste and debitage—and monitor the relative abundance of each category within various structures and inter-structural spaces to identify primary raw material storage units, workshops, and waste disposal areas. While the bone and antler tools identified at KSH form a key component of our broader zooarchaeological approach, it is important to note that this paper is not a detailed study of the site’s bone tool *technology*; rather it investigates the spatial distribution of bone tools as well as bone raw material and bone debitage to 1) independently test our previous interpretations of the function of some contexts (i.e., workshops or waste dumps), and 2) to identify the purpose of several of the aforementioned empty structures. Finally, we combine these observations with other architectural and artifactual indicators to monitor similarities and differences in the spatial organization of manufacturing activities across the excavated areas to examine the scale and extent of production activities and to explore the implication of these activities for social organization.

## Kura-Araxes economies

The human landscape of the KA oikumene is typified by small sites (>1–3 ha), but some larger towns and villages have also been identified, with a few anomalies such as KSH reaching 10 hectares or more [19,20]. The small size of most sites, low occupation intensity, and the presence of sites in rugged and seasonally inhospitable highlands, have been central to the description of many KA sites as temporary camps of mobile herders or small sedentary agropastoral communities [9,21–23]. These communities are often described as socially undifferentiated and egalitarian societies focused on subsistence farming and herding economies [18,19–29], because they lack evidence of specialized division of labor, centralized institutions, and social stratification typical of contemporaneous Mesopotamian cities or subsequent mountain societies of the second and first millennia BCE. These animal and farming economies were complemented with craft production and industrial activities. But who engaged in these practices and how manufacturing activities were organized require more attention. As the following examples show, there is little consensus on the answers to these questions, which range from household-based subsistence production to extra-household production specialization and division of labor.

Scholars who argue that KA economies were subsistence-based believe that craft production took place at the household level and was intended both for immediate use and some exchange. Borrowing from the work of Bourdieu, Wilkinson suggests that metallurgy and metal circulation in the KA world “may have had shared roots in…Neolithic moralities of communal exchange” [17], where gifting and a constant flow of valuable objects between kin served to generate social capital, designed to build and cement alliances. Unlike Wilkinson, Areshian [14] makes the case for the emergence of some degree of specialization and elite control of production in the early third millennium BCE. Areshian argues that the large hoard of bronze pickaxes and adzes found at the KA site of Jrashen (Armenia) exemplifies the use of copper and copper alloyed objects as “one of the principle means of wealth accumulation” [14]. He also sees evidence of specialized production at Mokhra Blur (Armenia), where large quantities of gromwell roots were laboriously processed to produce surplus red pigment, and at Shengavit (Armenia), where he argues that a metalworking workshop dating to the first half of the third millennium BCE operated a large-scale copper smelting and casting industry. Social hierarchy and labor specialization are hallmarks of the Uruk cities in Mesopotamia, and so for Areshian their presence in the KA world signifies “elements of urbanism” [14]. At the same time, Areshian acknowledges the importance of household labor. For example, he believes that one family at Mokhra Blur was responsible for pigment production.

In this sense, Areshian’s position is intermediary between those who characterize KA economies as largely subsistence-based and those who find clear evidence of specialized manufacturing economies. In the latter camp belong Stöllner and colleagues [30], who argue that a specialized division of labor was necessary for extracting, processing, and smelting gold at the late fourth–early third millennium BCE mine of Sakdrisi (Georgia) and the nearby KA settlement of Dzedzvebi. A similar argument is made by Hamon [31] about salt processing at Duzdaği (Nakhichevan), where architectural and artifactual indicators point to spatial segmentation of different activities across multiple salt processing operations. Stöllner used extensive experimental studies as well as the concentration of millstones in large workshops to show that the high labor and time investment needed for every step of gold extraction and processing required a specialized division of labor [30]. They further opine that the gold produced at Sakdrisi and Dzedzvebi promoted social differentiation through regional exchange [30]. But this point cannot be reconciled with overwhelming evidence for the absence of gold burial goods in KA graves and the absence of a wealth finance economy in the KA world. Nor is it explained what implications this specialized division of labor have for the social organization of the artisans’ communities, beyond a passing suggestion that these workers “did not pursue any agrarian activities” [30].

This diversity of viewpoints may reflect diversity in the social and economic structure of KA societies, but it may also result from the unsystematic approach to the study of the organization of production. Arguments for Neolithic ethos are largely based on the relatively light archaeological footprint of KA sites identified as small villages and temporary camps, but such an argument can also benefit from data that is recovered from larger villages and towns such as KSH. On the other hand, interpretations of specialized production, elite consumption of products, and social differentiation are either not entirely supported by evidentiary data, as is the case for Sakdrisi and Dzedzvebi, or are based on limited excavations and selective “circumstantial evidence” [14] such as at Mokhrablur and Jrashen. We contend that systematic investigations of the spatial layout of production activities at individual sites provide more reliable reconstructions of the organization of craft production and labor.

## Zooarchaeology and the spatial organization of production

The organization of production can be studied by reconstructing the various sites of production [18,32]—that is, the physical location of discrete manufacturing workshops and their constituent tasks and activities and the geographical relationship between them. Such a reconstruction provides critical insights into the organization of labor and labor specialization [33]. Specialization refers to the production of edible or “nonedible goods for consumption by social groups other than those that produced them” [34]. Specialization can take on many forms depending on the amount of time invested in production, the type of goods produced, and the organization of labor [33].

Among pre- or non-state-level societies, like those of the KA tradition, the household was the “backbone” of society and the most fundamental unit of economic output [35]. The primary economic function of households is domestic production. Depending on the size of households and the tempo of domestic activities, the mobilization of labor away from the immediate needs of that household toward non-subsistence-related activities like craft production can create scheduling conflicts within the household. Resolving these conflicts is a catalyst of social and economic change. Accommodating the labor needs of craft production may require some degree of division of labor and specialization that can disrupt the organization of households and create new opportunities for exchange between households or with other communities [35].

Systematic studies into the spatial organization of industrial practices and craft production rarely take faunal data into account. Faunal remains have been common raw materials for tool production throughout history and prehistory, because they were readily accessible and expendable materials that were subject to less recycling and curation than minerals or rare materials like metals or precious stones [36,37]. As such, the study of animal remains should be central to any comprehensive study of craft economies. Yet, beyond typo-technological studies of bone tools, zooarchaeology is rarely used in craft production studies. Zooarchaeology remains in large part the study of human subsistence practices in the past. Standard zooarchaeological measures, such as relative taxonomic abundance, can reliably reconstruct hunting and herding practices [25,38], while the application of animal body part representations and bone surface modifications to spatial analyses are used to reconstruct food provisioning and distribution mechanisms [25,39–44]. As we will demonstrate at KSH and as has been shown elsewhere [41], these same zooarchaeological measures are also versatile tools for understanding how people engaging in manufacturing activities organized their work space, what body parts of which animals they selected for tool making, how they stored and accessed their faunal raw material, where they made and used their bone tools, and ultimately where they discarded their waste.

## Köhne Shahar

**Fig 2:**
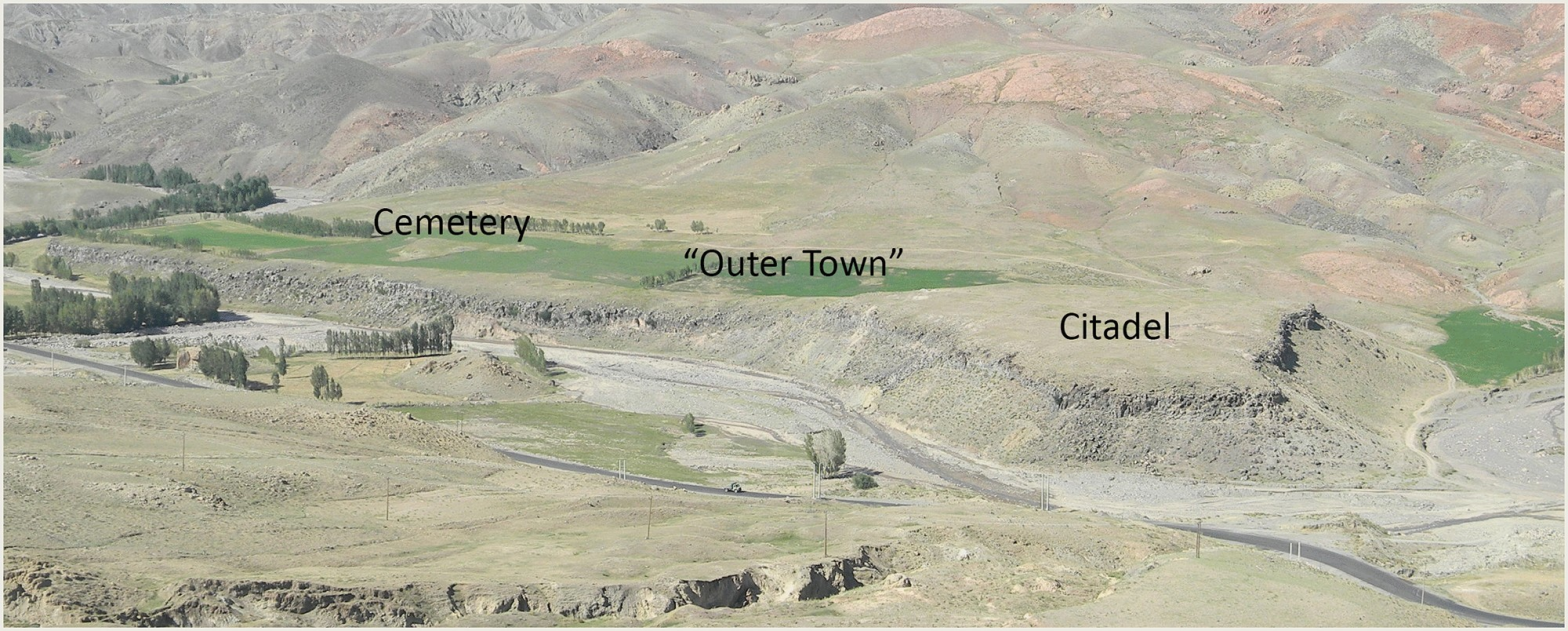
KSH and its three areas viewed from the south-southwest.

**Fig 3:**
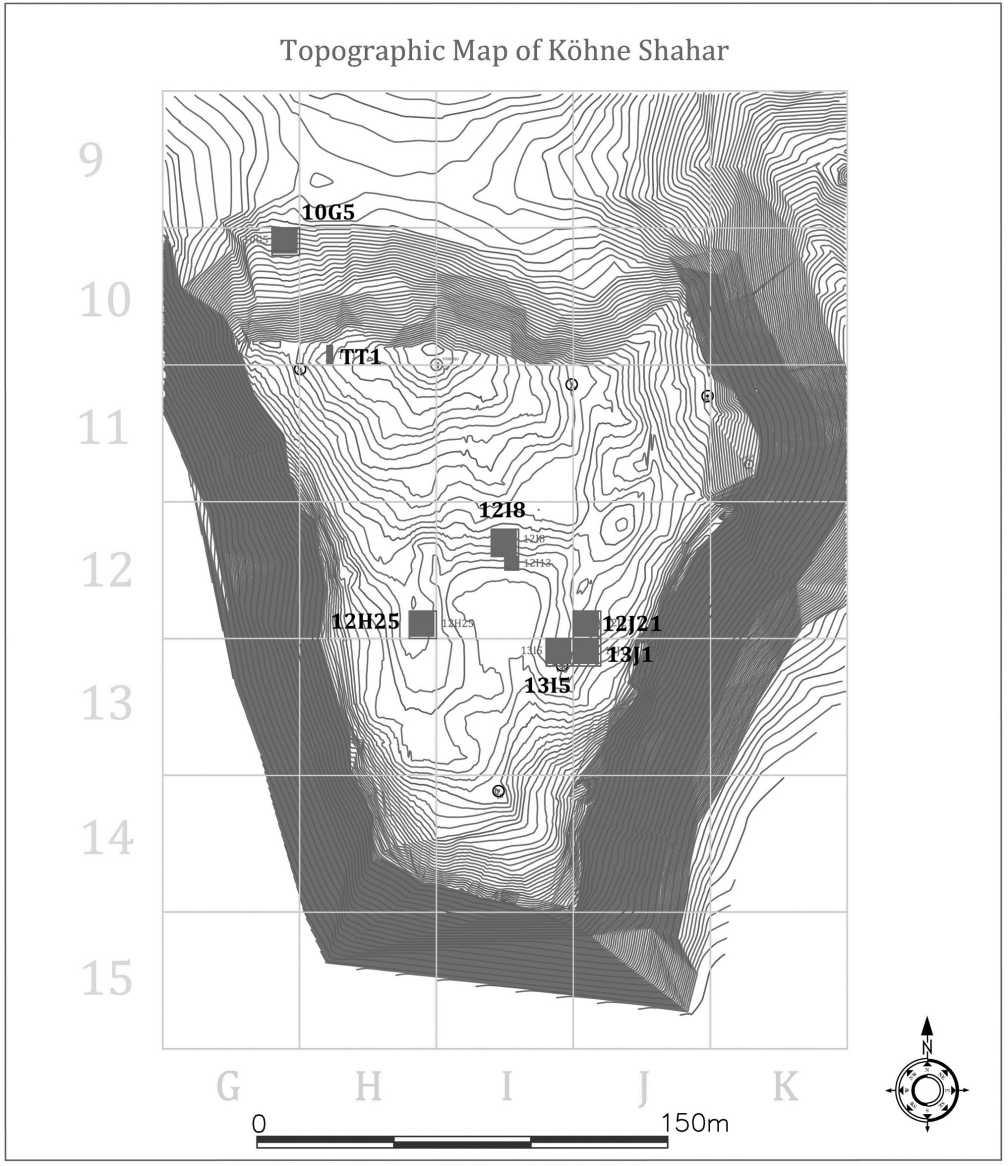
Trench locations in each neighborhood superimposed on a topographic map of the citadel. The grey outline of the wall separating the citadel from the “outer town” can be seen along the northern edge of the citadel.

**Fig 4:**
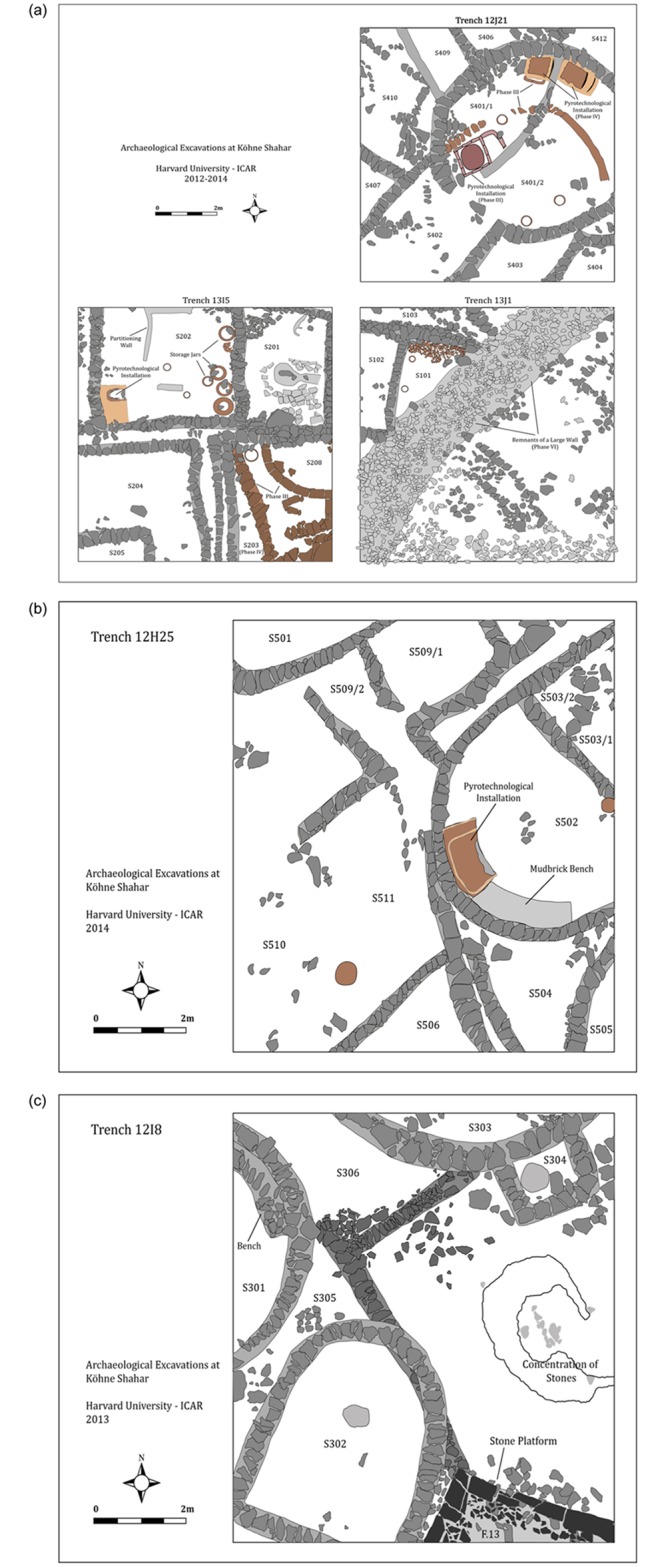
a) Schematic drawing of the eastern neighborhood (ENH). Phase III is depicted using the color brown, Phases IV–V are depicted using grey. The outline of a large curvilinear structure from the disturbed final Phase VI was partially detected in Trench 13J1. The drawing was prepared by Karim Alizadeh using AutoCAD. b) Schematic drawing of the western neighborhood (WNH). The drawing was prepared by Karim Alizadeh using AutoCAD. c) Schematic drawing of the central neighborhood (CNH). Walls of the possible Phase IV are depicted using a darker shade of grey. The drawing was prepared by Karim Alizadeh using AutoCAD.

Köhne Shahar (KSH) is located at 1905m asl near the town of Chaldran in the West Azerbaijan Province of northwestern Iran (Fig 1). With an area of approximately 15 hectares, KSH is one of the largest sites in the KA world [45]. It consists of a citadel, separated by a large wall from an “outer town” and a cemetery (Fig 2). The citadel comprises of several discrete neighborhoods divided by radial alleyways leading to a central plaza (Fig 3) [45]. Stratigraphy of the citadel, which is approximately 250m^2^ in areas, consists of five distinct phases of occupation, numbered I through V from oldest to newest. Phases I–III date to ca. 3200–2800 BCE, and Phases IV–V date to ca. 2800–2500 BCE [46,47]. A disturbed cultural layer on the surface of the citadel primarily consists of structural foundations of stone, resembling layouts of typical KA circular buildings. This disturbed cultural layer was not detected in our excavations. Thus, we assume that there was a sixth architectural phase in the citadel (for example see plan of Trench 13J1 in Fig 4a) that can only be understood by future investigations. The stratigraphic relationship between the citadel and the extramural areas remains unknown, but ceramics and architectural analyses show that at least part of the final occupation of the “outer town” was contemporaneous with Phases IV–V of the citadel [47].

Between 2012 and 2014, Karim Alizadeh and his team excavated five 10 × 10m trenches in the eastern (ENH; Trenches 12J21, 13J1,13I5), central (CNH; Trench 12I8 and 12I13), and western (WNH; Trench 12H25) neighborhoods inside the citadel (Fig 3). All recovered materials from these excavations, including faunal remains, are curated at the Cultural Heritage Organization of West Azerbaijan Province in Urmia, Iran. Underneath the aforementioned disturbed surface deposits, excavations exposed *in situ* cultural deposits and numerous round and rectilinear structures and inter-structural spaces in Phases IV and V (Fig 4a–4c) [13]. Part of a small curvilinear structure belonging to Phase III was also excavated in Trench 13I5 in ENH (Fig 4a). Some of these contexts contained a wealth of primary and secondary evidence for craft production. Four of the structures—S202 (ENH), S401/1 (ENH), S401/2 (ENH), and S502 (WNH)—contained similarly-sized small pyrotechnological installations as well as numerous tuyères, beads and bead blanks, and large hammerstones, all suggesting that these structures were primary manufacturing workshops [13]. One structure (S101 in ENH) and one inter-structural space (S306 in CNH) were most likely primary waste disposal areas, as they contained thick secondary deposits of ash, broken tools, broken ceramic vessels and wasters, broken crucibles, and slags. Numerous other spaces and structures of various sizes were also interspersed among these workshops and waste disposal areas. These “empty” structures were largely devoid of any notable features and artifacts and contained little evidence for either manufacturing or domestic activities, and so their functions remain unclear. The results of an extensive geophysical survey pointed to a high frequency of manufacturing activities throughout the citadel [13]. While this does not mean that all areas of the citadel were dedicated solely to craft production, the spatial scope of activities suggests that craft production was practiced throughout the community.

**Fig 5:**
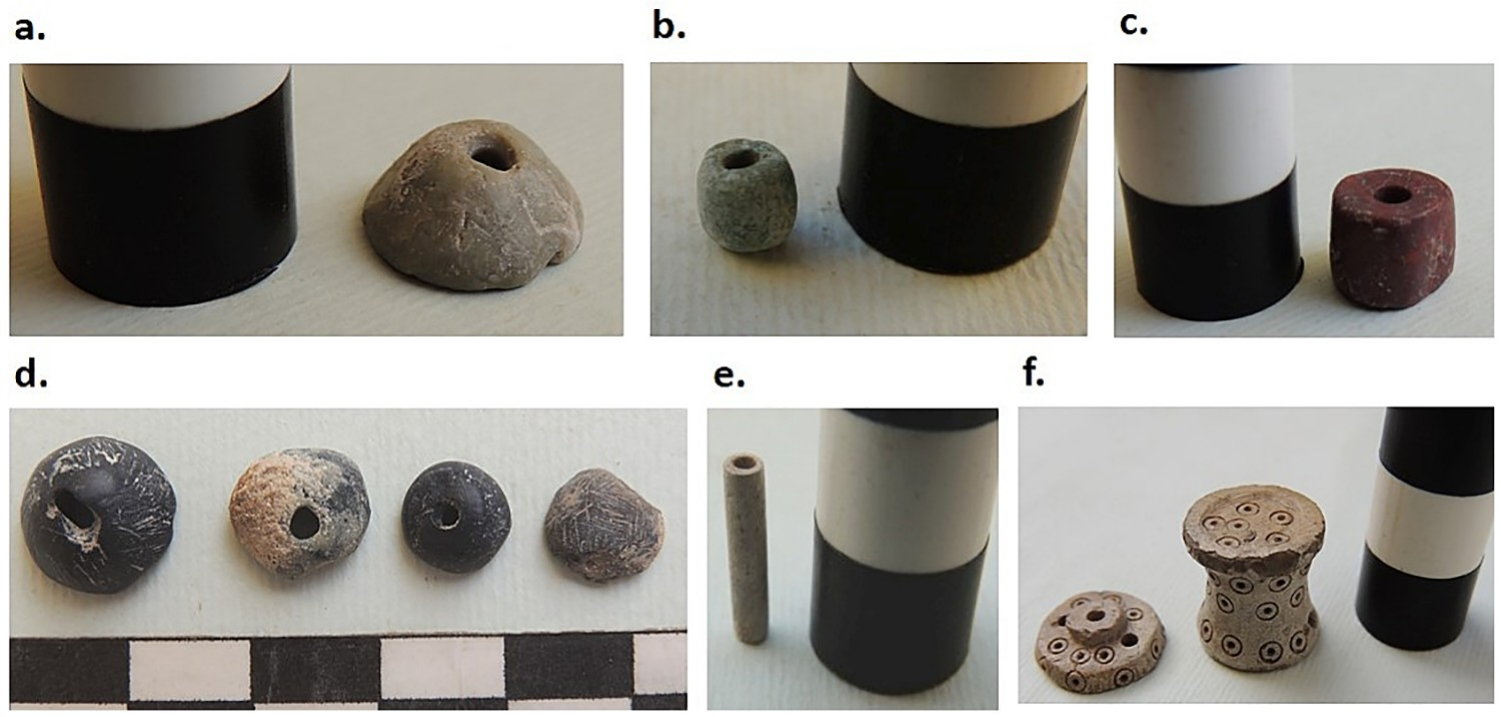
Beads and bead blanks from S401/2 in ENH (a–c) and S502 in WNH (d–e); miniature container from S502 in WNH (f). Specimens photographed by Karim Alizadeh.

The full range of objects produced at KSH is not yet known. The abundance of stone and soapstone beads and bead blanks (Fig 5a–5e), the small size of the pyrotechnological installations, and the small size of the crucibles and slags suggests that manufacturing focused on the production of small, possibly ornamental objects [13]. Finished objects are rare at the site, but the plethora of beads, a miniature container from S502 (WNH) possibly made of steatite (Fig 5f), and a silver ring from the fill of a disturbed grave in the cemetery of KSH may exemplify the type of objects manufactured inside the citadel.

In addition to stone beads, bone and antler are the most abundant classes of worked remains at KSH. Recovery of complete bone and antler tools, used and broken tools, and relatively large quantities of unshaped antler in contexts containing stone bead blanks, pyrotechnological installations, hammer-stone implements, and manufacturing waste deposits, as well as some of the aforementioned “empty” structures, indicates that bone and antler raw materials were cached and then modified into tools that were then used to manufacture other finished objects. Thus, our primary goal in this study is to identify the loci of bone and antler raw material storage (storage units), loci of bone tool production and use in manufacturing other objects (workshops), and loci where broken and consumed tools and bone and antler debitage were discarded (waste dumps).

## Methods

Discrete cultural layers at KSH that were identified by changes in sediment color and texture or segregation by architectural remains (e.g. walls) were excavated in 10cm arbitrary spits. Sediments were sieved using 5mm and 2mm mesh screens. Zooarchaeological data at KSH was recorded in the field in the 2015 and 2016 study seasons from all faunal remains and across all contexts. All necessary permits were obtained for the described study, which complied with all relevant regulations. Data recording took place near the site at the Qara Kelisa (St. Thaddeus) Monastery located in Chaldran County in northwestern Iran. This space and permission to study the remains were furnished by the Iranian Center for Archaeological Research, which is a research and administrative subdivision of the Iranian Cultural Heritage Organization and the primary agency responsible for issuing permits for archaeological research.

Studied remains include handpicked finds, as well as finds collected during sieving. For each specimen in the identifiable fraction of the assemblage various taxonomic, anatomical (element, bone portion), demographic (age, sex), and taphonomic variables were recorded in an Excel spreadsheet. Taxonomic identification was based on a digital comparative collection generously provided by Mary Stiner and Natalie Munro, as well as several published manuals [47–51]. Bones were identified to the smallest possible taxonomic category; specimens that could not be securely identified taxonomically were assigned to body size categories. Established morphological protocols were also used for sex and age determination [48,52–55]. Taphonomic categories that were recorded include cutmarks [56], sawmarks, fragmentation [57], burning [58], and animal gnawing. Whenever specimen completeness permitted, identified bones were measured following von den Driesch [59].

In this paper a two-step approach is applied to study the faunal remains within individual neighborhoods and their constituent structures and extramural spaces. The first step explores variation in the distribution of animal skeletal parts to separate faunal patterns most likely associated with manufacturing activities from non-manufacturing related refuse, to pinpoint neighborhoods, structures, and spaces where bone tool production and use took place. Because of variability in their mechanical properties (e.g., strength and flexibility), certain skeletal elements of certain animals (e.g. deer antler) [60] are more commonly selected for tool manufacture than others. The recovery of relatively large quantities of antler and cattle horncore from some of the contexts at KSH suggests that they may have been particularly critical to manufacturing. To better examine their role, body parts are combined into three anatomical categories—horn/antler, head (skull and mandibles, excluding teeth), and body (axial and appendicular skeletons)—and their abundances are examined relative to their frequencies in an ideal animal using the number of identified specimens (NISP) [61].

In the next step, contexts associated with bone raw material storage, bone tool use, and bone debris disposal are identified. This step investigates the spatial distribution of modified and unmodified skeletal elements in the areas flagged to be of interest in step one. Each of the three stages can be identified by the relative proportions of raw materials, production waste like debris, and finished tools. Statistical differences in the spatial distribution of these remains will be determined using Chi-squared (χ^2^) test of independence. Raw material storage and primary manufacturing areas are signaled by a predominance of raw materials (e.g., unmodified bones, antler, and horn). Manufacturing areas also yield high proportions of modified specimens, namely tools, while storage and waste disposal areas yield a higher proportion of unmodified specimens. Raw materials suitable for tool manufacture include large unmodified bone pieces with the appropriate qualities for tool manufacture. Debris refers to unmodified pieces of raw materials or debitage too small to be shaped into tools. Waste areas may also contain consumed or broken tools. The relative proportions of modified and unmodified specimens will be measured using NISP.

Because raw materials must be larger on average than finished tools and tools are larger than manufacturing debris, the average length and size range of fragments provide an additional line of evidence to define the different stages of bone tool production. We measured each specimen along its longest axis and recorded it in millimeters. Certain elements like horncore and antler are identifiable even when highly fragmented. Post-depositional fragmentation and accidental post-recovery breakage can create extremely small, yet identifiable fragments of bone from even the largest pieces [62]. Inclusion of these small fragments in our analyses may mask the presence of raw material and artificially inflate the abundance of waste debris. To reduce the role of fragmentation on these measurements, analyses do not include specimens smaller than 2cm, and those broken during and after excavation.

It is important to note that this is not a technological study. Worked bones were identified as part of zooarchaeological data collection of the complete faunal assemblage, and their taxonomic, anatomical, and taphonomic variables were recorded just as they were for unworked specimens. As was discussed above, taphonomic variables that were recorded include unique codes for the type, size, location, and intensity of several types of intentional bone modifications. The presence of sawmarks, perforations, and obvious anthropogenic alterations to the natural shape of certain body parts, were among the key criteria used to distinguish worked bone and bone tools from unworked faunal remains.

For this study, we combined the data from Phases IV and V because in large trenches the stratigraphic boundary between the two phases is not clearly distinguishable, and certain walls and structures were in continuous use in both phases [46]. Except for the intermural Public Space (PS) in CNH, all discrete contexts—structures and spaces—are identified by the letter “S”, followed by a unique three-digit number (Fig 4a–4c). Other than S208 and S209 (Trench 13I5) in ENH Phase III (Fig 4a), all other contexts belong to Phases IV and V. S402 (Trench 12J21) and S103 (Trench 13J1) (Fig 4a), which were excavated in different seasons, are likely parts of the same structure and so their contents are combined and analyzed together and identified as S103.

## Results

**Fig 6:**
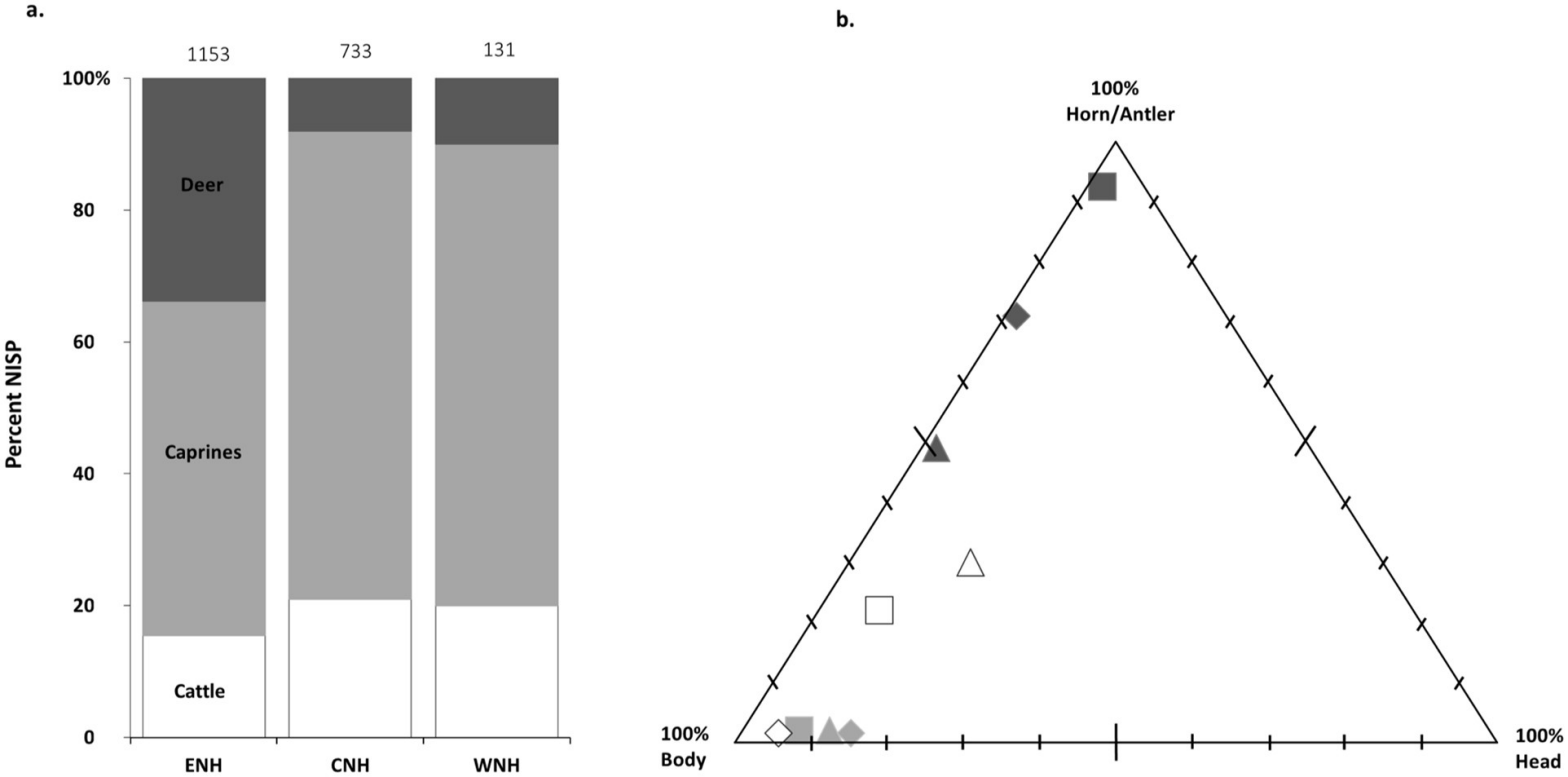
Relative abundance of cattle, caprines, and deer bones (a) and body part representation of cattle, caprines, and deer in each neighborhood (b). Square: ENH, triangle: CNH, diamond: WNH. Numbers at the top of each bar represent the total number of ungulate bones identified in that neighborhood; colors in the triplot correspond with that for each species in the bar graph.

In total, 3143 specimens were identified from all neighborhoods. The majority of these (NISP = 2017) belongs to sheep and goat (Caprinae), domestic cattle (*Bos taurus*), and red or fallow deer (Cervidae) (Fig 6a). The most striking pattern in species representation is the numerous deer specimens (NISP = 387) in ENH, where they account for 34% of ungulates, compared to 7–11% in the other neighborhoods (Fig 6a). An examination of ungulate body-part representation immediately reveals that antler is significantly over-represented in all three neighborhoods (Fig 6b). This is especially striking in ENH, where it represents 92% of all deer specimens. Antler also accounts for 49% and 71% of deer specimens in CNH and WNH respectively. Horncore is also well-represented, accounting for about 30% of cattle elements in ENH and CNH. Because of their inflated representation and utility as raw materials, antler and horn core are central to the remaining analyses.

Sixty caprine, cattle, and deer specimens were modified into tools (Table 1), nearly half of which (n = 29) were recovered in ENH. Of these, 62% (n = 18) were made of antler. Most of the worked specimens in ENH were identified in Phase IV–V, where antler tools (n = 14) account for 67% of the worked faunal remains in that phase. In contrast, antler tools are rare in CNH, where 81% of the worked remains (n = 27) are bone. Fifty-four percent of the worked bone specimens at KSH are made of caprine ulnae (n = 19) (Table 2), most of which (n = 13) were recovered in CNH. Eighty-six percent of these worked bones are awls or perforators, but also include an awl shaft made of a caprine metatarsal (CNH), and two spindle whorls made of the femur head of a large bovids like cattle, one each in ENH IV–V and CNH (Table 2). The antler tools primarily consist of pointed tools such as needles and bobbins that may have been used for weaving (Table 3).

**Table 1:** Bone and antler tool and worked specimen counts for each neighborhood.

Tissue Type	Bone	Antler	Total
ENH III	4	4	8
ENH IV–V	7	14	21
ENH subtotal	11	18	29
CNH	22	5	27
WNH	2	2	4
Total	35	25	60

**Table 2:** Anatomical and typological categories of bone tools and worked specimens for each neighborhood.

Neighborhoods	ENH III	ENH IV–V	ENH subtotal	CNH	WNH	Total
*Body Part*						
Femur	-	1	1	1	-	2
Metapodial	2	2	4	2	-	6
Phalanx	-	1	1	-	-	1
Radius	-	-	-	3	-	3
Rib	-	-	-	1	-	1
Tibia	1	-	1	2	-	3
Ulna	1	3	4	13	2	19
Total			11	22	2	35
*Type*						
Awl/Perforator	4	5	9	19	2	30
Handle	-	-	-	1	-	1
Pendant	-	1	1	-	-	1
Spindle whorl	-	1	1	1	-	2
Other	-	-	-	1	-	1
Total			11	22	2	35

**Table 3:** Typological categories of antler tools and worked specimens for each neighborhood.

Neighborhoods	ENH III	ENH IV–V	ENH subtotal	CNH	WNH	Total
Awl/Perforator	1	2	3	4	2	9
Bobbin	-	7	7	-	-	7
Comb	1	-	1	-	-	1
Needle	2	4	6	-	-	6
Other	-	1	1	1	-	2
Total	4	14	18	5	2	25

Faunal remains were not uniformly distributed across all contexts. To reduce sample size bias, only those spaces and structures with a minimum NISP of 25 were investigated further. Twelve of the 27 contexts in ENH and five of the seven contexts in CNH met this criterion (S1 Appendix). WNH is not presented in detail because of its small samples (NISP = 249), but notable observations from WNH are incorporated into the discussion.

### Deer antler

**Fig 7:**
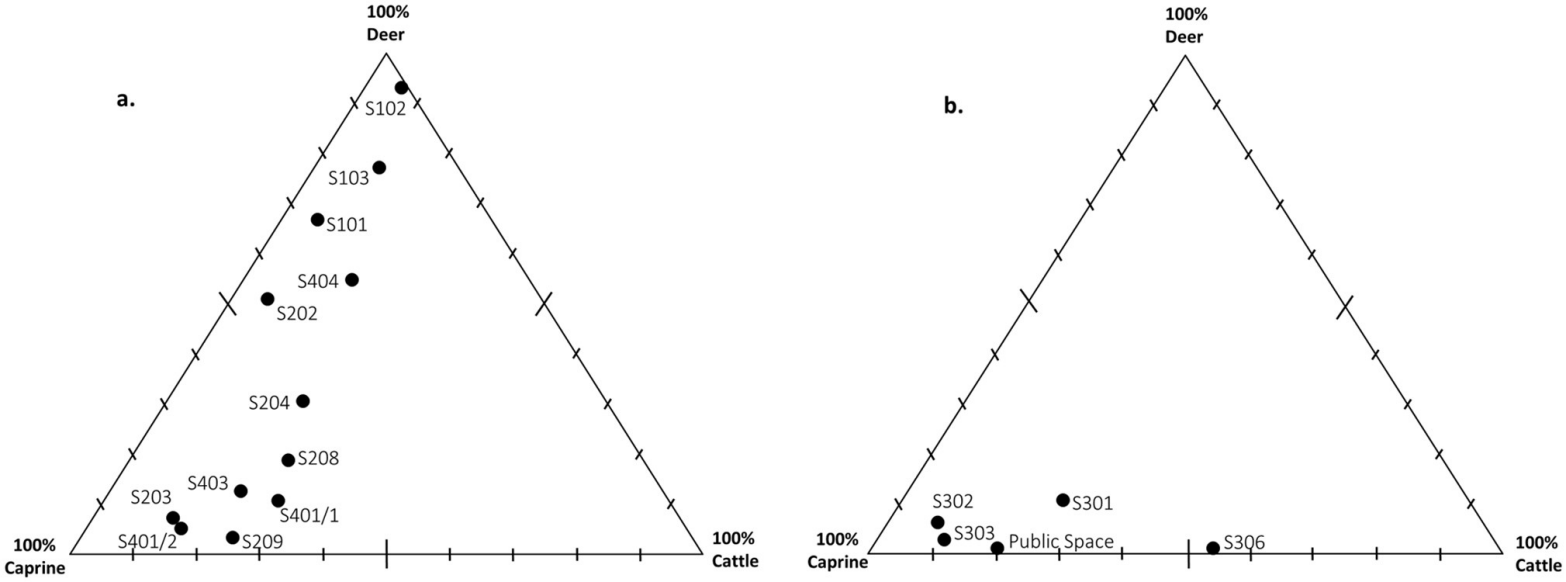
Relative abundance of cattle, caprines, and deer bones within individual structures and spaces in ENH (a) and CNH (b).

**Fig 8:**
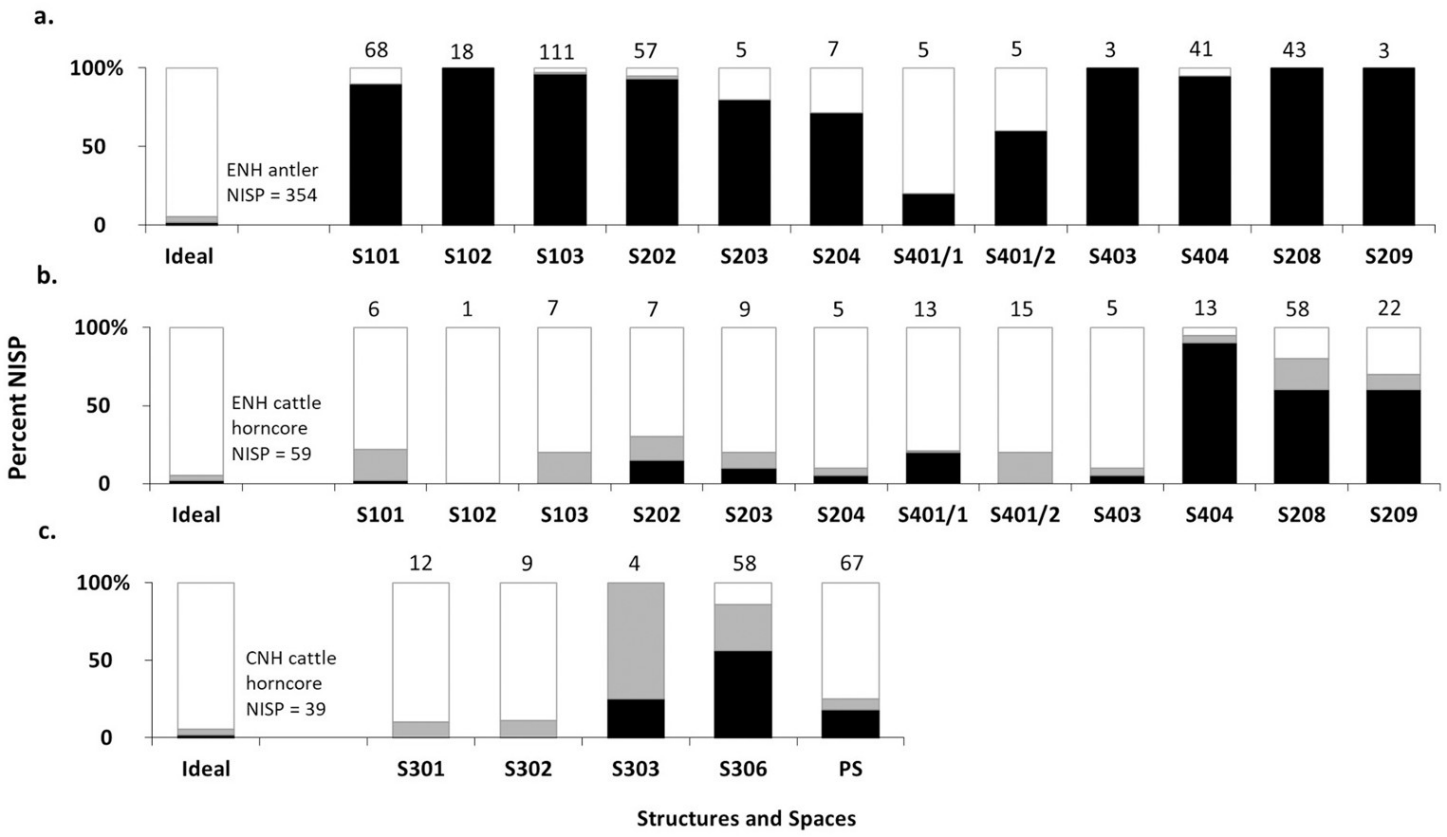
a) deer body part representation in ENH; b) cattle body part representation in ENH; c) cattle body part representation in CNH. Black: antler/horncore, grey: head, white: body. Data are compared to the percentage of each anatomical region in a complete or ideal individual. Numbers at the top of each bar represent the total number of deer or cattle bones in that context.

**Fig 9:**
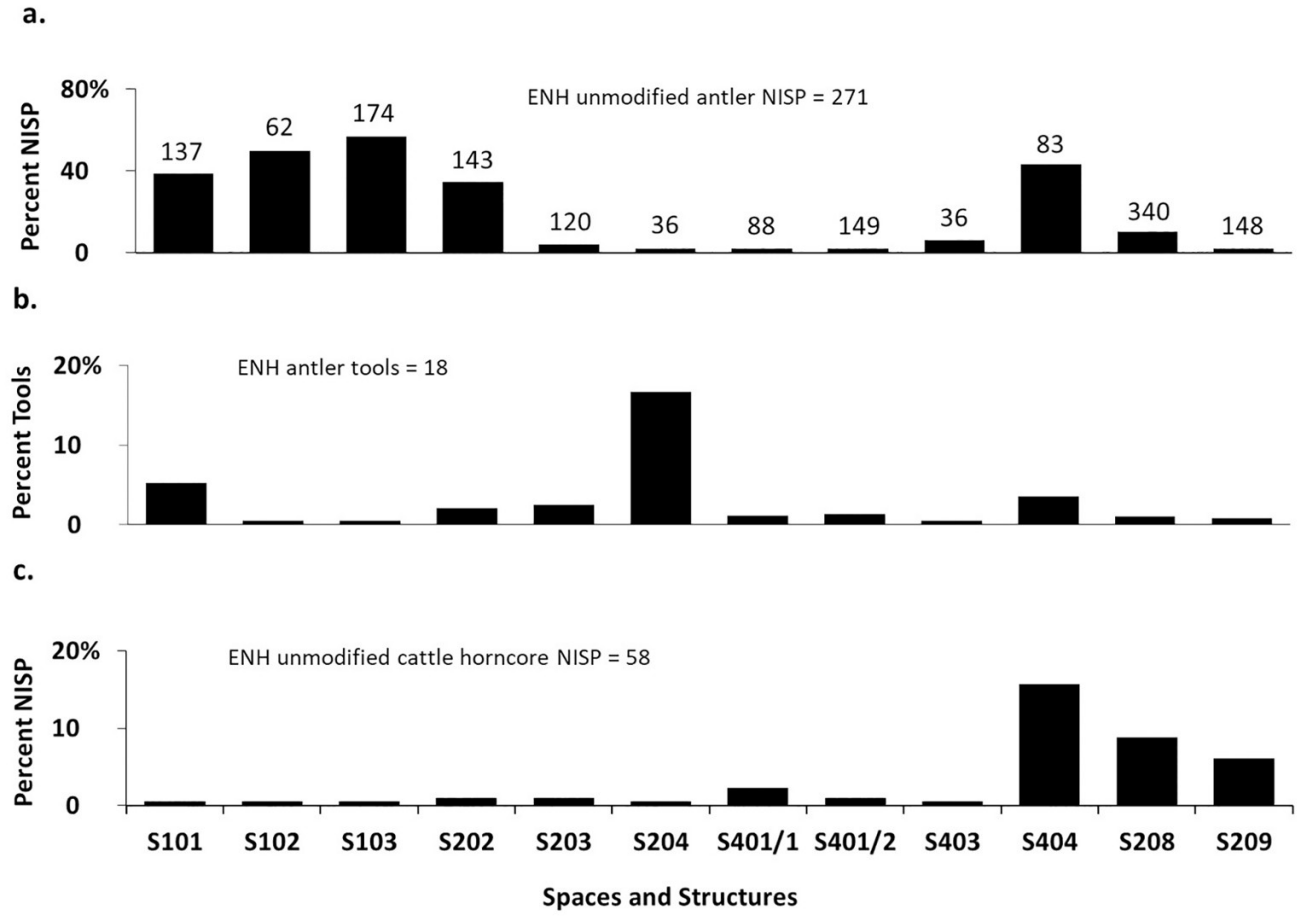
a) percentage of unmodified antler fragments in ENH; b) percentage of antler tools in ENH; c) percentage of unmodified cattle horncore fragments in ENH. Numbers at the top of each bar represent the total number of identified bones (NISP) in that context.

**Fig 10:**
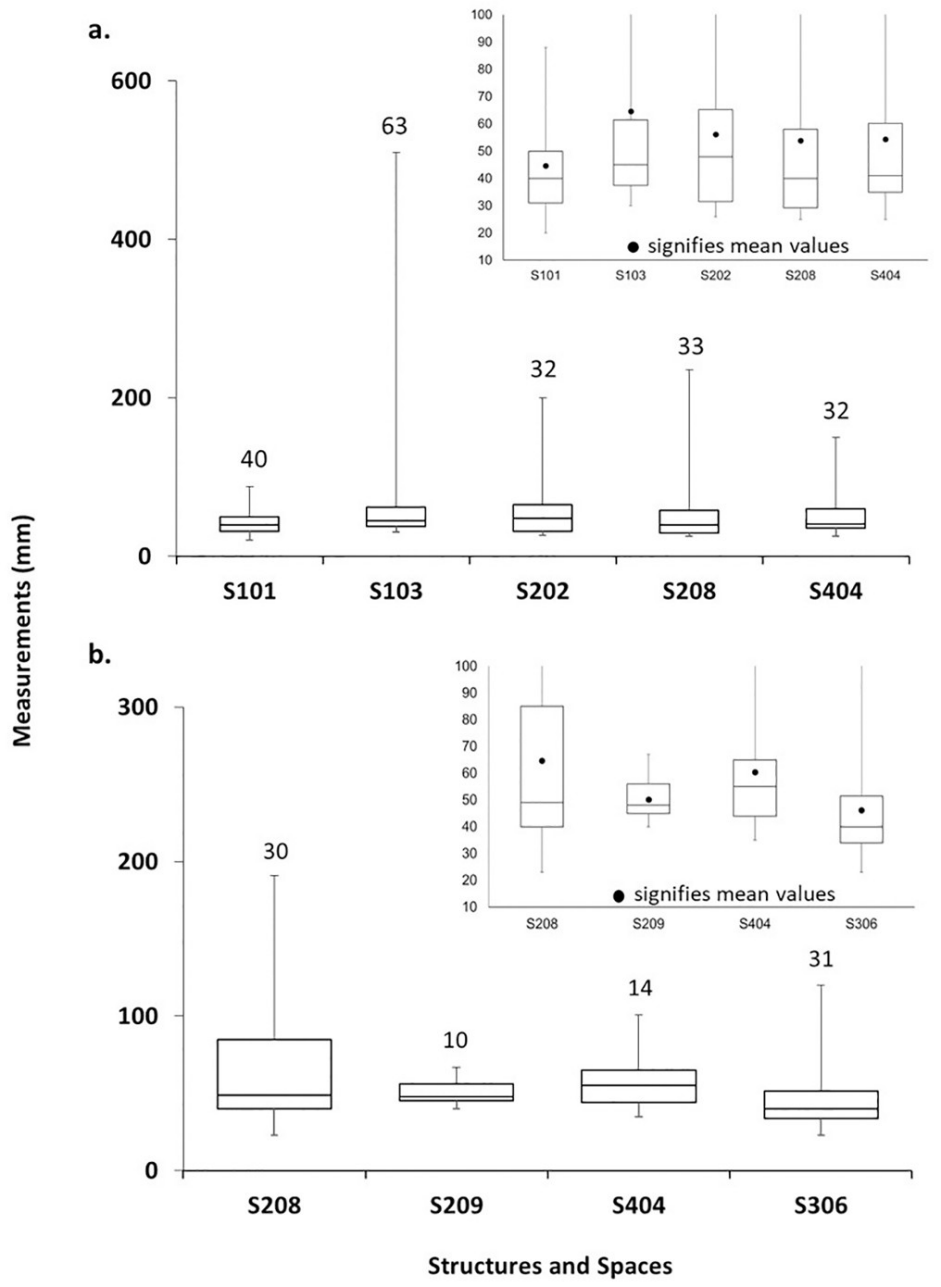
a) antler fragment size distribution in ENH; b) horncore fragment size distribution in ENH and CNH. Numbers at the top of each bar represent the number of specimens measured in each context.

**Fig 11:**
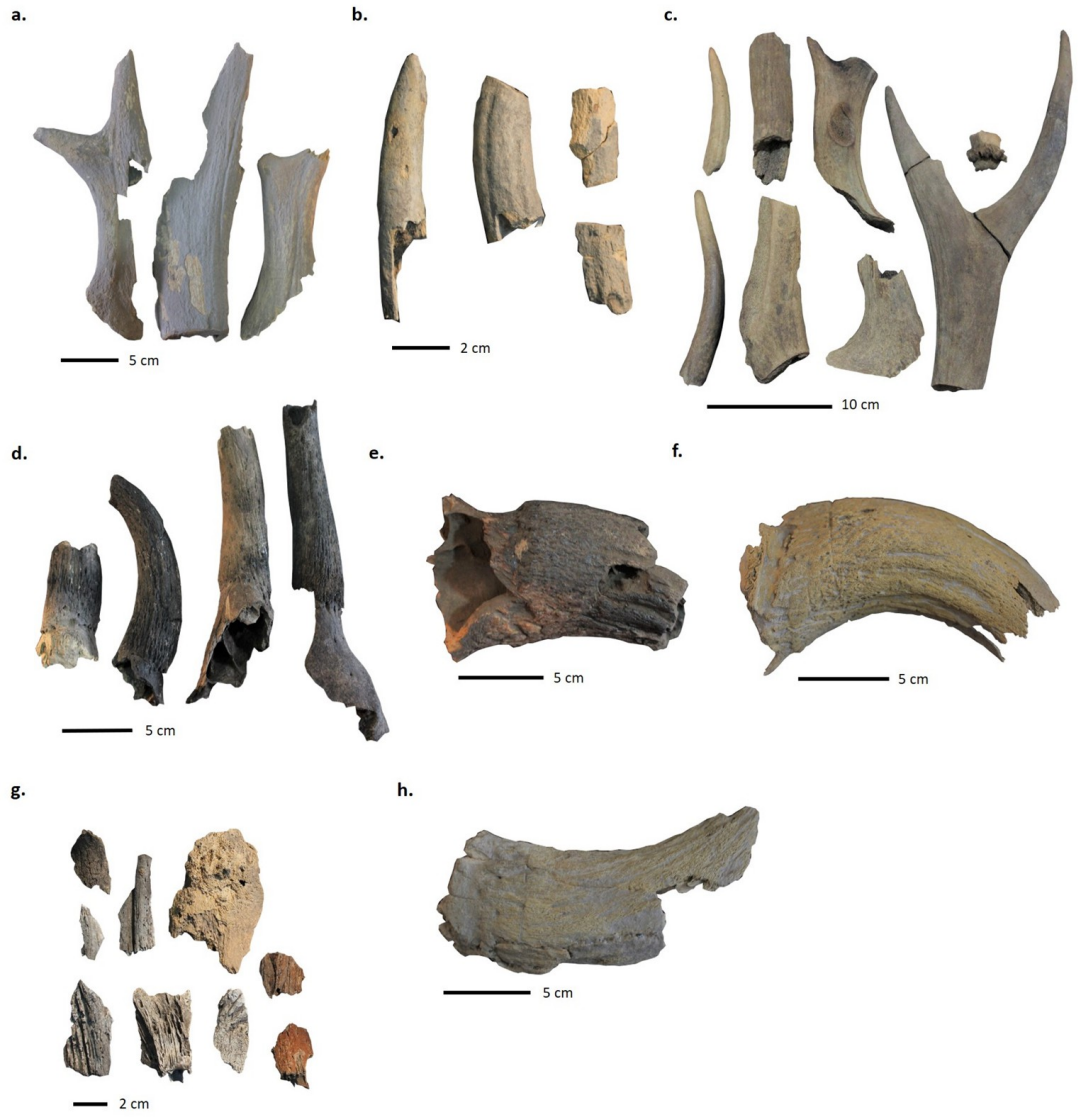
Antler specimens from S103 in ENH IV–V (a), S101 in ENH IV–V (b), and S208 in ENH III (c); cattle horncore specimens from S208 in ENH III (d), S306 in CNH (e, g), and the Public Space in CNH (f); wild goat horncore from the Public Space in CNH (h). Note long cutmarks at the base of the cattle and wild goat horncores from the Public Space (f, h). Specimens photographed by Siavash Samei and Karim Alizadeh.

In ENH, deer remains are dominant in five contexts and present in small amounts in seven others (Fig 7a). Antler is the most common deer body part in all 12 contexts, except S401/1 (Fig 8a). Unmodified antler is most concentrated in S101–S103, S202, and S404, where it accounts for 40–75% of all recovered bones, followed by S208, where it accounts for 10% of all bones (Fig 9a). Average fragment size is small in most contexts, and for the most part tightly distributed (Fig 10a). The widest range of fragment sizes, and the largest bone fragments are found in S103 (Figs 10a and 11a). In contrast, the most tightly distributed and smallest fragments are found in S101 (Figs 10a and 11b). Fragment size distributions in S202, S208 and S404 are intermediate between the two, as are their mean and median values (Figs 10a and 11c). The sample size for S102 is too small for fragment size analysis. As was mentioned, the majority of worked specimens in ENH are antler tools (Table 1). Antler tools are most common in S204, where they comprise 20% of all recovered bones (Fig 9b). Antler tools and blanks were found in lower frequencies in S101, S202, S203, S208, and S404. One antler tool was also found in S401/1 and two were recovered from S401/2. Differences in the spatial distribution of unmodified antler and these modified specimens are statistically significant (χ^2^ [15, N = 363] = 60.245, p<.001), indicating that antler raw materials and tools are concentrated in different contexts.

### Cattle horncore

Cattle are present in all 12 contexts in ENH but are not dominant in any of them (Fig 7a). Horncore is the most common cattle body part in S208, S209, and S404 (Fig 8b), where it is also more highly concentrated (Fig 9c): horncore comprises 6–16% of all recovered remains in these contexts compared to, 0–2% in other contexts. More abundant large fragments in S208 have widened and skewed the size distribution of horncore fragments (Figs 10b and 11d). The distribution of fragment sizes was much tighter, and mean values were much lower in S209. Size distribution and mean values are intermediate in S404.

**Fig 12:**
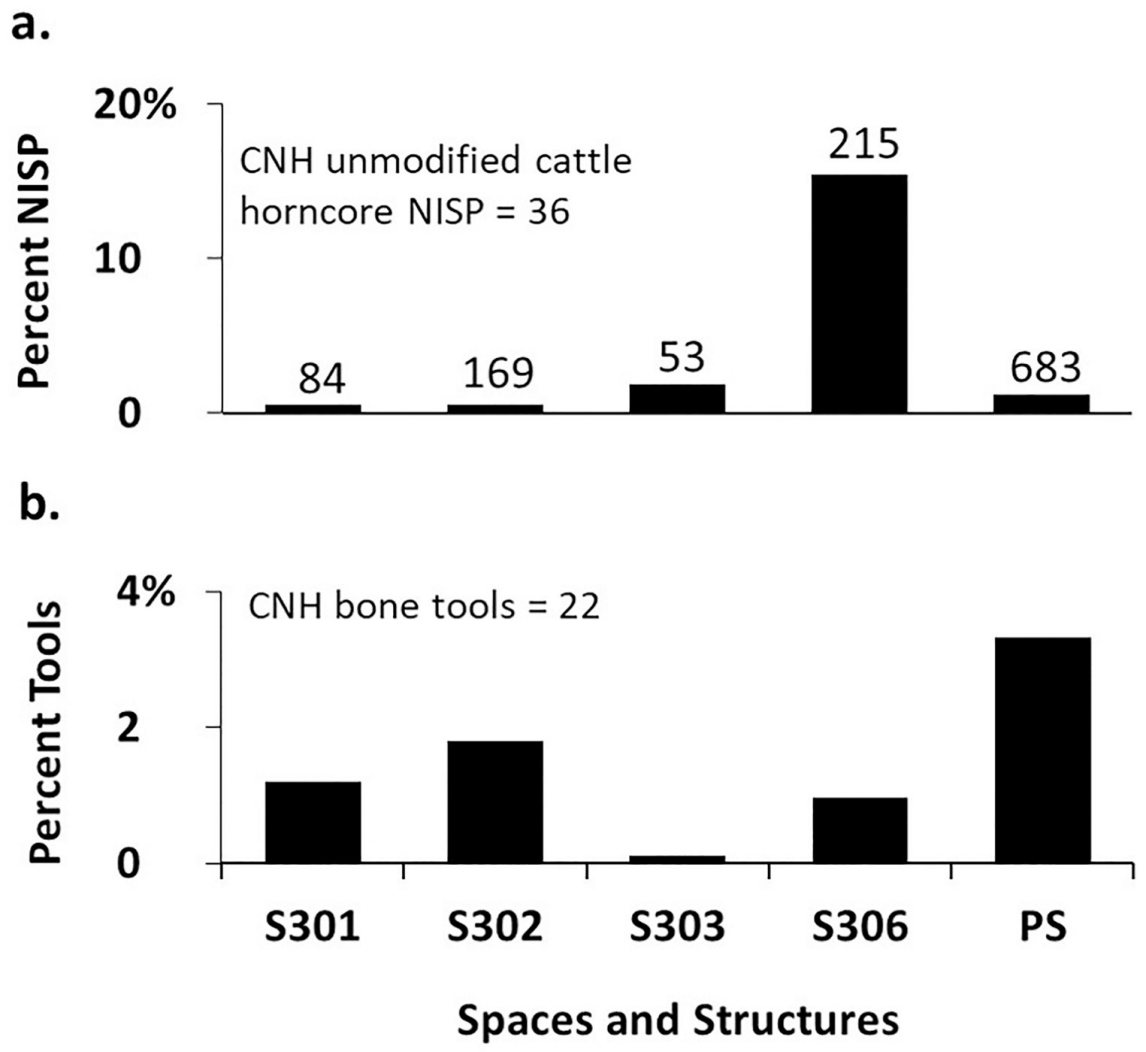
a) percentage of unmodified cattle horncore fragments in CNH; b) percentage of bone tools in CNH. Numbers at the top of each bar represent the total number of identified bones (NISP) in that context.

Cattle is present in all five contexts in CNH, but it is the most common taxon only in S306 (Fig 7b), where horncore is the most common and most abundant cattle body part (Figs 8c and 12a). Horncore is also present, but in lower percentages in S303 and the Public Space (PS). Horncore specimens in CNH are highly fragmented, although two large specimens, one of which has basal cutmarks, were found in the Public Space and S306 (Fig 11e and 11f). Sample size is only large enough for fragment size analysis in S306, so comparison between different contexts within CNH is not possible, but because of the abundance of small horncore fragments, mean and median horncore fragment sizes in S306 are lower than in S209 (Figs 10b and 11g). The majority of the 22 bone tools in CNH were found in the Public Space and S302, where they respectively comprise 4% and 2% of all identified finds (Fig 12b). One bone tool was recovered from S301, and two others were found in S306. Statistically significant differences in the spatial distribution of cattle horncore and bone tools (χ^2^ [4, N = 61] = 58.95, p<.001), suggest that they were part of two different processes.

## Discussion

High concentrations of finished antler tools in S204 and the presence of antler tools in S202, S203, and S401 in ENH IV–V suggest that these structures were workshops where primary manufacturing took place. This observation confirms the pattern already detected from abundant non-faunal contextual and artifactual evidence for manufacturing activities in some of these structures [13]. This is also true of S502 in WNH, where two bone tools were found in association with a similar suite of workshop-related features and artifacts. The association of these antler and bone implements with other contextual and artifactual indicators are discussed in more detail in the next section.

Among the other, non-manufacturing structures, high percentages of unmodified antler and horncore, as well as antler and horncore fragment size distribution and skewness values indicate that some of these were storage and waste disposal areas. S102 and S103 in ENH IV–V contained high percentages of unworked antler, including several large pieces (Fig 11a). Low concentrations of other body parts, and an absence of features, tools, and other notable artifacts in these “empty” structures, suggest that they were primarily used for storing raw materials.

Combined lines of evidence from S101 in ENH IV–V, S209 in ENH III, and S306 in CNH show that these spaces were used as waste disposal areas. S101 contained a high percentage of small pieces of unmodified antler and antler blanks (Fig 11b), while S209 and S306 contained a large quantity of fragmented cattle horncores (Fig 11g). In each case, animal remains were intermixed with thick secondary deposits of industrial-grade ash from oven clean-ups. S101 and S306 also contained slags and numerous broken crucibles. Open spaces such as S403 in ENH IV–V and S510 in WNH likely played similar roles as waste dumps, because each contained a broken bone tool and several small antler fragments embedded in secondary ash deposits.

The functions of S404 in ENH IV–V, S208 in ENH III, and 505 in WNH are less clear because of limited archaeological exposure, and so our interpretations are tentative. In all three, the absence of waste debris like slags and ash rules out their use as waste disposal areas. In S505, only six unmodified antler specimens were found. The absence of workshop-related features like pyrotechnological installations and worked materials like tools, suggests that S505 was a storage unit. Intermediate antler fragment sizes for S208 and S404, intermediate horncore fragment sizes for S404, and a few antler tools in S404 suggest that they may have been manufacturing areas. However, like S505, the absence of tools and workshop-related features, and the high percentages of unworked antler and cattle horncore are more indicative of storage. Certainly, it is possible that both raw material caching and manufacturing activities were taking place in these spaces.

How does the distribution of different tools and raw materials correlate with workshop activities? What do the bone tools reveal about the type of objects that were manufactured and the nature of manufacturing activities in each workshop?

### Workshop activities

**Fig 13:**
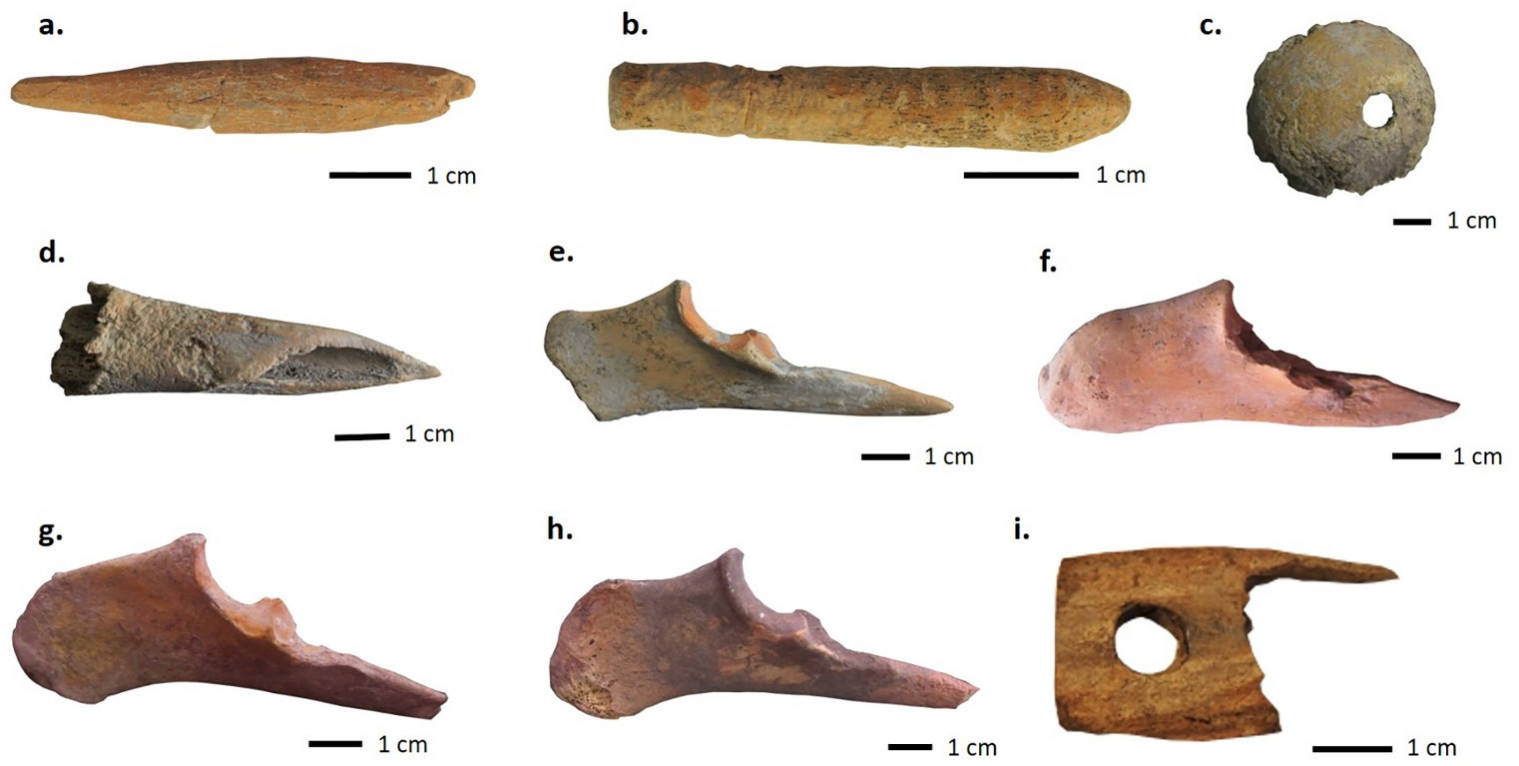
Several examples of bone and antler tools at KSH. a) possible double-pointed antler bobbin from S204 in ENH IV–V; b) possible antler spindle shaft fragment from S202 in ENH IV–V; c) bone spindle whorl from S202 in ENH IV–V; bone awls from 401/2 in ENH IV–V (d), S502 in WNH (e), S302 in CNH (f), Public Space in CNH (g), and S306 in CNH (h)—note striations on the tip of awls from S401/2 (d) and S502 (e); antler shearing comb from S208 in ENH III (i). Specimens photographed by Siavash Samei and Karim Alizadeh.

**Fig 14:**
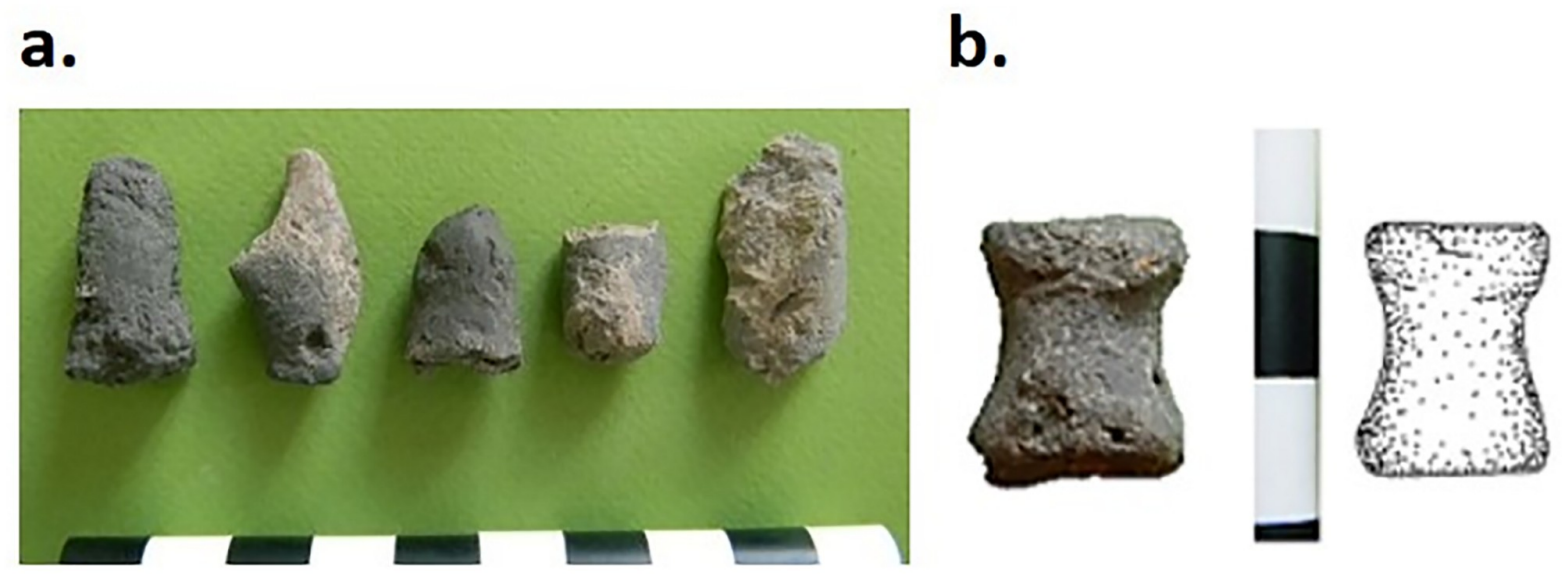
a–b) clay objects, possibly administrative tokens or loom weights from S101 in ENH IV–V. Specimens photographed by Karim Alizadeh.

ENH IV–V features two antler storage units, and two waste disposal areas, and several manufacturing units. S202–S204 were most likely mixed workshops where a range of production activities took place, including weaving. Twelve of the 18 antler tools in ENH are concentrated in S202–S204 and resemble fragments of long weaving needles and possible shuttle bobbins (Fig 13a and 13b). Needles can range between 2–5mm in diameter, and between 70–100mm in length [63]. The bobbins are pointed on both ends and lack flanges, which suggests that they were incorporated into shuttles that carry threads of weft yarn when weaving with a loom [64]. Additional evidence for textile production includes a conical spindle whorl made of a cow femur head in S202 (Fig 13c). Several artifacts from S101 (Fig 14a and 14b), which are typically considered to be administrative clay tokens [13], have elsewhere been identified as loom weights [15]. The length and girth of needles and bobbins, and the size and weight of spindle whorls depend on the type of fabric and the method of weaving. The spindle whorl found in S202 (Fig 13c) is broken and so could not be accurately measured, but its small size (ca. 25mm radius) and light weight (19.2g) suggest that it was best suited for working with a loosely spun fabric [15]. The small size and gracile build of the bobbins and needles supports this observation. At the moment it is unclear whether plant or animal fabric was spun in these workshops. However, elsewhere we have shown that a proportion of the sheep and goats herded at KSH were kept into adulthood, providing residents with access to wool and hair [16,25].

In addition to antler tools, five bone awls were also found in ENH IV–V, one of them in S401/2. Striations around the circumference of the awl tips (e.g. Fig 13d) were created by perforation. The association of these awls with soapstone beads and bead blanks suggests that they were used for bead manufacturing in S401/2. 401/2’s twin structure S401/1, lacked any direct evidence for textile or bead production. Both had similar pyrotechnological installations, but S401/1 also contained two ceramic tuyères, numerous large and heavy stone implements and hammerstones, and a copper awl. Aside from one small and flat antler implement, S401/1 does not contain any bone or antler tools. This, and the fact that access from S401/1 to antler storage unit S103 was blocked by the internal subdivision between the twin facilities (Fig 4a), hint at specialization in space and activities in the two halves of S401.

In WNH, S502 contains large heavy hammerstones, ceramic tuyères, and a pyrotechnological installation that resembles those located in S202 and S401. Yet, the low concentration of antler in WNH suggests that antler was not central to its activities as it was in ENH IV–V. Evidence for the production of soapstone and steatite objects is most prominent in WNH, where several perforated beads and bead blanks (Fig 5d and 5e) were found in association with four bone awls, including one in S502 (Fig 13e), with perforation use wear on their tips. Use-wear analyses are needed to confirm our hypotheses about the function of these bone awls in S401 and S502.

Antler is also nearly absent in CNH, where a large number of bone awls were found. Horn-working was the dominant activity in CNH. Sheath removal took place in the Public Space, where some large horncore pieces and several smaller fragments including one with basal cutmarks (Fig 11f) were found. Horn-working waste was then removed and deposited in S306. In addition to bull horn, one mouflon and nine bezoar horncore fragments were also identified in S303, S306, and in the Public Space, including one with basal cutmarks (Fig 11h). How the bone awls, most of which are made of sheep-goat ulnae (Fig 13f–13h), related to horn-working is unknown. The bone tools were primarily used and discarded in and around the Public Space, including in S301 and S302, where a groundstone quern and ceramic vessels most resemble kitchenware [13]. This suggests that these tools were used in the domestic activities taking place around the Public Space.

Although further excavations are needed at KSH for a systematic study of temporal continuity in craft production, our evidence presents some tentative but interesting patterns of continuity. S208 in ENH III is located just underneath S203 in ENH IV–V and contains several uncut cattle horncore fragments, antler needles, antler raw material, and an antler comb that is typically used to clean and disentangle fleece [64] (Figs 11c, 11d and 13i). This suggests that workshop activities in general, and horn-working and weaving in particular, endured at KSH for at least two phases of occupation.

To summarize, our zooarchaeological analyses not only confirmed previous interpretations of some structures and spaces as primary workshops and waste disposal areas [13], but identified the nature of some activities in several other structures and areas for the first time. These include two antler storage areas and several other possible workshops and/or storage spaces. Zooarchaeological revelations about these heretofore unidentified areas, coupled with previous archaeological observations and the geophysical survey, make a compelling argument for the widespread distribution of manufacturing workshops and associated activities across three neighborhoods of the fortified settlement [13]. What does this community-wide craft economy suggest about the social organization of the settlement? Although at this moment we cannot determine whether each workshop represents an independent manufacturing unit or a stage within a longer and more complex chaîne opératoire, the available data points toward a non-stratified, heterarchical social organization within the citadel.

### Social organization

There are important differences in the type of production activities and utilized raw materials across this community: notably exclusive evidence of textile working using antler tools in one part of ENH IV–V and horn working in CNH. There are also important differences in the architectural layout between and within neighborhoods; from rectilinear layout of S202–S204 and the two-chambered curvilinear layout of S401 in ENH IV–V, to the single-chambered curvilinear structures of WNH and CNH. The exact reason behind these architectural differences is at present unknown. But what is clear is that neither the differences in manufacturing activities and raw material types, nor in the architectural layouts denote any type of vertical or stratified relationship in the organization of labor. The absence of a rigid social hierarchy within the excavated areas is also reflected in uniformity in the furniture, material culture, and waste of various workshops. Most workshops have similar-sized small pyrotechnological installations, small crucibles, and similar production waste like ash and small-sized slags. This uniformity suggests that despite exclusive production of certain goods in certain areas, different workshops also produced a mix of similar, possibly small ornamental objects, which included stone beads.

Further evidence for the absence of an entrenched social hierarchy is provided by the fact that no one structure stands out as a central or elite unit dedicated to labor coordination or control of manufacturing activities. Indeed, compartmentalization of space into primary workshops, storage areas, and waste dumps, and the close proximity of these areas to one another served as a logical means for the workers to organize their space, rather than a top-down control of access to raw materials or management of specific tasks. Evidence suggests that one waste disposal area directly served one or a pair of workshops, and antler storage units in ENH IV–V were directly accessible from S401/2 and S202–S204. Lack of a top-down system to control access to raw materials is not surprising insofar as faunal remains are concerned. The long bones of sheep and goat were not precious or valuable materials and would have been easily accessible thanks to the site’s well-developed animal husbandry practices [16,25]. Ease of access explains the widespread distribution of worked and unworked long bones throughout the excavated areas. Antler was also easily accessible. Deer body part representation shows that KSH residents actively hunted large deer but also collected shed antler whenever possible [25].

The absence of a stratified social order, top-down elite control of production, and restricted access to resources does not mean that KSH was a socially undifferentiated, Neolithic-like society. In Near Eastern archaeology, strict hierarchies and social stratification have disproportionally influenced theorizing about social complexity [65–67]. In this light, the descriptions of KA communities as Neolithic-like or socially undifferentiated are emblematic of a linear and urban-centric narrative of social evolution that conceives of Mesopotamian cities as the apex of complexity, thereby dismissing their non-urban contemporaries in the highlands as relics or reminders of a simpler bygone era. This is both a mischaracterization of Neolithic societies and the concept of social complexity. Complexity can also be defined in terms of heterogeneity, lateral modes of organization, and heterarchy [68–70].

Heterarchy is defined as the “relation of elements to one another when they are unranked, or when they possess the potential for being ranked in a number of different ways” [65]. Heterarchy does not refer to a specific type of social structure [69], and this indeterminacy is its strength [70], because it allows for heterarchy to be used as an abstract descriptor of a wide range of social relationships without adhering to reductionist or at times dichotomous labels such as egalitarian or hierarchical [71]. Heterarchies have two key features: 1) horizontal organization and differentiation, and 2) flexible hierarchies [72].

The evidence, as laid out above, affirms the horizontal organization of economic production at KSH. Heterarchy can often be found in societies with community-level craft economies in which household is the base unit of production [72,73]. The intermixture of manufacturing activities with habitation areas suggests that household members were the primary laborers in the workshop units at KSH. Previous zooarchaeological studies have highlighted the significance of households to the site’s economy. Animal husbandry at KSH has been shown to follow a herd security management strategy [25], which is typical of household-based agropastoral economies [74–76]. We have also demonstrated that household members that worked and resided in the three neighborhoods accessed animal products directly either by raising their own animals or through intra-site exchange [25]; and not indirectly through a third party or elite apparatus, as is often seen in strictly hierarchical or socially stratified societies [39].

This point about direct food provisioning also highlights the importance of lateral differentiation and exchange at KSH. Intra-site exchange in horizontally-organized communities, especially those with community-level craft economies is a critical risk buffering strategy, particularly in environmentally unpredictable areas [72,77]. The exact relationship between the excavated workshops is still not clear, but there are two possibilities: either each workshop was a self-contained unit of production, or each of them was part of longer, more complex operational chains. Either scenario points to a horizontal, laterally differentiated and interdependent craft economy that encouraged exchange and movement of goods between, for example, those engaging in textile production, horn working, or the manufacture of other objects. The concentration of antler in ENH IV–V is another indicator of exchange. KSH residents actively hunted deer for both meat and antler, but deer remains are not equally represented in all contexts [25]: high concentration of antler in ENH and other deer bones in CNH is a sign of the movement of different deer body parts within the community. This lateral interdependence allows different elements within the settlement to focus on the manufacture of certain goods, or on the completion of certain tasks, thereby reducing the possibility of scheduling conflicts between individuals or between and within households. Risk minimization was not limited to the craft economy of KSH, and was also a key food production strategy, given the unpredictable nature of the Mediterranean climate of the region in the fourth and third millennia BCE [25,78,79].

Finally, we should note that the horizontal decision-making lattice of heterarchies does not preclude the presence of social rankings or flexible hierarchies; only the development of entrenched and centralized hierarchies [80,81]. In heterarchies, different sources of power are counterpoised among different institutions, elements, and individuals [80], with no one element enjoying hegemonic control over power sources [77]. The horizontal and differential distribution of power is not reflected in monumental or palatial structures, as we would expect in socially stratified urban societies, but is rather embodied by the type of lateral differentiation discussed earlier [72, 82]. These power sources may have also been implicated in coordinating the construction and maintenance of such communal projects as the large stone wall of the citadel [47]. We will however postpone a more in-depth discussion about multiple sources of power at KSH until several outstanding issues are resolved in further excavations. Two of these issues are the nature of possible administrative activities in ENH IV–V [13], and the nature of the relationship between the residents of the citadel and those inhabiting the extramural area.

### KSH and Kura-Araxes economies

The large size and make up of KSH make it a unique site in the KA cultural tradition [5,14,45]. Nevertheless, its overall evidence for craft production fits well within the broader context of cultural and economic development in the KA world. The economies of the first KA communities that appeared in the mid-fourth millennium BCE in the southern Caucasus were largely characterized by small-scale, household-based subsistence economies [16,25–27]. These economies underwent a major shift in the early third millennium BCE, a period that is contemporaneous with the habitation of KSH IV–V. In this period, the first KA settlements were abandoned and new settlements were founded in formerly unoccupied areas within the southern Caucasus [83,84]. This shift was accompanied by the appearance of a mosaic of local ceramic styles that replaced the more uniform material culture of the earlier KA tradition [83].

Some of these new settlements were founded near copper and obsidian sources [83]. Evidence for craft production at the aforementioned sites of Mokhra Blur (pigment production), Duzdaği (salt mining), Shengavit (copper production), and Sakdrisi and Balitshi-Dzedzvebi (gold mining and production), all also date to this later phase of the KA ceramic horizon [14,30,31,83], as does KA expansion out of the southern Caucasus and the appearance of KA communities and occupations across the Zagros mountains and Anatolia. Zooarchaeological data from Godin Teppe VI (Iran) and Arslantepe VI B (Turkey) point to specialized sheep and goat management for wool and hair production, which is corroborated by the stone and bone tool technologies and, at Arslantepe, by textile fragments [15,16,85].

As discussed earlier, interpretations of the organization of craft production in the KA world may not all reliable, but the collective evidence, despite all of its shortcomings, points to an economic mosaic in the early third millennium BCE in which different communities focused on the extraction of different materials and the manufacture of different goods.

Economic diversification and heterogeneity across the KA world resulted in lateral differentiation that promoted exchange between different KA communities. The scale of production at KSH suggests that exchange was probably not limited to intra-site interactions and that its manufactured goods were also traded and exchanged with other communities in the region. This lateral differentiation in regional economies is a hallmark of what Antonio Sagona called the KA heterarchy [86]. Evidence for the KA heterarchy is further provided by the absence of rigid hierarchies and political centralizations. Indeed, despite some possible regional two-tier settlement hierarchies [14], no one KA settlement can be claimed as the political heart of the tradition. Instead, KA communities were largely characterized by horizontal decision-making processes [86].

Much remains unknown about the KA world, but new studies and excavations, and reassessment of earlier works in light of fresh data and theoretical advances can greatly enhance our understanding of the social and economic organization of this highland tradition. In a period in ancient Southwest Asia when the impressive archaeological footprint of urbanism can overshadow evidence of cultural diversity and complexity in the “marginal” highlands, our observations contribute to a broader, non-urban-centric examination of the markers of social complexity in the ancient Near East and a more nuanced narrative of life in the mountains.

## Supporting information

S1 Appendix The number of identified specimens (NISP) for all discrete contexts (structures and spaces) in the eastern (ENH) and central (CNH) neighborhoods. Only those contexts with a minimum NISP of 25 were included in this study. Theses contexts are highlighted in bold. NISP for S103 also includes specimens found in S402; the two contexts were excavated in different seasons and in two adjacent trenches, but are most likely parts of the same structure.
(XLSX)
